# Impact of Long‐Term Use of Anticoagulants Among Elderly Patients Undergoing Left Atrial Appendage Occlusion Procedures in the United States: A Retrospective Cohort Study

**DOI:** 10.1002/hsr2.70812

**Published:** 2025-04-29

**Authors:** Jasninder Singh Dhaliwal, Samay Mehta, Manraj S. Sekhon, Arush Rajotia, Haresh Gandhi, Kavin Raj, Jarmanjeet Singh, Mushood Ahmed, Renuka Verma, Hemamalini Sakthivel, Kamleshun Ramphul, Raheel Ahmed, Swaiman Singh

**Affiliations:** ^1^ Department of Internal Medicine University of California Riverside California USA; ^2^ Birmingham Medical School, College of Medical and Dental Sciences University of Birmingham Birmingham UK; ^3^ Department of Cardiology University of California Riverside California USA; ^4^ Rawalpindi Medical University Rawalpindi Pakistan; ^5^ Department of Internal Medicine Kirk Kerkorian School of Medicine at UNLV Las Vegas Nevada USA; ^6^ One Brooklyn Health System/Interfaith Medical Ctr Program Brooklyn New York USA; ^7^ Independent Researcher Triolet Mauritius; ^8^ National Heart and Lung Institute Imperial College London London UK; ^9^ Cardiovascular Diseases. Mayo Clinic Minneapolis Minnesota USA

Atrial fibrillation (AF) is the most common sustained arrythmia worldwide, with its global prevalence rising from 33.5 to 59 million between 2010 and 2019 [1]. AF contributes to a significant proportion of ischemic strokes which are associated with worse clinical outcomes compared to non‐AF strokes. Long‐term anticoagulation is a well‐established therapy for stroke prevention in AF, however its use is often limited by factors such as bleeding risk, side effects and noncompliance. Left atrial appendage occlusion (LAAO), which targets the primary site of thrombus formation, has emerged as an effective alternative in these patients, with the added potential of reducing all‐cause mortality [2]. Both surgical and percutaneous approaches are available for LAAO. However, procedure‐related and in‐hospital complications for LAAO still remain a concern, and therefore there is a need for further patient exploratory analyses to identify factors associated with reduced risks. This study specifically compares complication rates between elderly patients not on long‐term anticoagulation (more typical candidates for LAAO) and those on long‐term anticoagulation before the procedure. We hypothesized that prior background anticoagulation may impact LAAO outcomes due to its potential influence on physiological profiles and thrombotic/bleeding risks.

Based on recommendations from prior studies, patients with a primary diagnosis of AF/flutter and undergoing percutaneous LAAO procedures were extracted from the 2016–2021 National Inpatient Sample (NIS). Cases aged younger than 60 were excluded. The sample was divided into patients with and without long‐term (current) use of anticoagulants (ICD‐10 code “*Z79.01*”) and baseline patient characteristics were compared. A propensity‐score matched (PSM) sample was generated, at a 1:1 ratio, at a caliper of 0.01 to balance our two cohorts. To accurately assess the differences in outcomes between the two PSM groups, we constructed several multivariable regression models. Our selection of variables in these models were guided by the results from the univariable regression analyses, where variables with a *p*‐value less than 0.20 were eligible for inclusion. For each outcome, we also calculated adjusted odds ratios (aOR) with corresponding confidence intervals (CIs). We maintained our statistical analyses at a *p*‐value of less than 0.05 (2‐sided). All the statistical analyses were performed via SPSS 29.0 and STATA 18.0. As this study utilized deidentified publicly available data from the NIS without patient identifiers or interaction, no institutional review board approval and additional patient consent was required.

In our pre‐PSM cohort, of the 131,445 patients meeting the selection criteria, 72,200 (54.9%) had a history of long‐term (current) anticoagulation use. Differences in baseline characteristics were observed as patients with long‐term anticoagulant use reported a higher prevalence of hypertension (45.9% vs. 44.4%, *p* < 0.01), dyslipidaemia (65.5% vs. 61.5%, *p* < 0.01), smoking status (42.0% vs. 35.8%, *p* < 0.01), prior PCI (17.8% vs. 16.2%, *p* < 0.01), prior stroke (23.2% vs. 21.9%, *p* < 0.01), and obesity (17.9% vs. 16.3%, *p* < 0.01) while having fewer cases of diabetes (33.8% vs 35.0%, *p* < 0.01), chronic kidney disease (22.5% vs. 23.2%, *p* < 0.01), liver cirrhosis (1.6% vs. 2.0%, *p* < 0.01), and COPD (16.5% vs. 18.3%, *p* < 0.01). Both groups were predominantly male, white and covered by Medicare (Table 1). For our PSM‐matched cohort, this involved 55,225 patients with and 55,225 without long term anticoagulant use. Statistical differences in the prevalence of COPD, drug abuse, obesity, alcohol abuse, liver cirrhosis, PVD, history of PCI, history of CABG, diabetes, hypertension, insurance forms, and weekend admissions were not present. However, age, sex, race, dyslipidemia, smoking, CKD, prior MI and prior stroke had statistical difference as shown in Table 1.

**Table 1:** Characteristics and complications of patients aged ≥ 60 with versus without long‐term anticoagulant use admitted for left atrial appendage occlusion procedures in the United States.

	Pre‐PSM sample			PSM sample		
	Patients not on long term anticoagulant (*n* = 59,245) (%)	Patients on long term anticoagulant (*n* = 72,200) (%)	*p*‐value	Patients not on long term anticoagulant (*n* = 55,225) (%)	Patients on long term anticoagulant (*n* = 55,225) (%)	*p*‐value
Mean age	76.69	76.66	0.496	76.73	76.26	< 0.01
Weekend admissions	0.6	0.5	0.096	0.5	0.5	0.537
Sex			< 0.01			< 0.01
Male	57.3	59.1		58.0	56.4	
Female	42.7	40.9		42.0	43.6	
Primary payer form			< 0.01			0.586
Medicare	89.5	90.0		89.7	89.8	
Medicaid	0.9	0.7		0.8	0.8	
Private	7.7	7.3		7.6	7.5	
Race			< 0.01			0.015
White	88.0	89.8		88.9	89.0	
Black	4.1	3.5		4.0	3.7	
Hispanics	4.5	3.8		4.2	4.2	
Hypertension	44.4	45.9	< 0.01	44.8	44.7	0.672
Dyslipidemia	61.5	65.5	< 0.01	62.9	63.5	0.031
Smoking	35.8	42.0	< 0.01	36.9	37.6	0.012
Diabetes	35.0	33.8	< 0.01	34.6	34.6	0.950
Chronic kidney disease	23.2	22.5	< 0.01	22.6	23.2	0.035
History of CABG	13.4	13.3	0.592	13.3	13.3	0.929
History PCI	16.2	17.8	< 0.01	16.6	16.8	0.242
Peripheral vascular disease	7.4	7.1	0.156	7.3	7.3	0.954
Prior stroke	21.9	23.2	< 0.01	22.3	21.7	0.035
Liver cirrhosis	2.0	1.6	< 0.01	1.7	1.7	0.245
Alcohol abuse	1.3	1.2	0.190	1.3	1.3	0.287
Prior MI	12.5	12.5	0.752	12.3	13.1	< 0.01
Obesity	16.3	17.9	< 0.01	16.6	16.9	0.083
Drug abuse	0.3	0.3	0.806	0.3	0.3	1.000
COPD	18.3	16.5	< 0.01	17.4	17.8	0.097
**Complications**						
Pre‐PSM sample				PSM sample		
Sepsis	0.2	0.1	< 0.01	0.14	0.05	< 0.01
Intra/postoperative hemorrhage hematoma	0.5	0.4	< 0.01	0.5	0.4	< 0.01
Pericardial effusion	3.0	2.8	0.013	3.0	2.9	0.131
Cardiac arrest	0.14	0.08	< 0.01	0.14	0.09	0.025
Acute kidney injury	2.5	1.9	< 0.01	2.5	2.0	< 0.01
Acute ischemic stroke	0.2	0.2	0.065	0.2	0.2	0.294
Acute myocardial infarction	0.2	0.1	< 0.01	0.2	0.1	< 0.01
Cardiogenic shock	0.2	0.1	0.014	0.13	0.10	0.179
Died	0.2	0.1	< 0.01	0.2	0.1	< 0.01

*Note:* This presents baseline characteristics and the complications of patients aged ≥ 60 with versus without long‐term anticoagulant use admitted for left atrial appendage occlusion procedures in the United States.

Abbreviation: PSM, propensity score matching.

In addition, according to the PSM sample, patients with long‐term anticoagulation reported fewer complications such as sepsis (aOR 0.37, 95% CI 0.243–0.564, *p* < 0.001), intra/postoperative hemorrhage/hematoma (aOR 0.757, 95.5% CI 0.632–0.906, *p* = 0.002), cardiac arrest (aOR 0.673, 95% CI 0.470–0.964, *p* = 0.031), acute kidney injury (aOR 0.784, 95% CI 0.722–0.851, *p* < 0.001) and acute myocardial infarction (aOR 0.404, 95% CI 0.285–0.573, *p* < 0.001). No differences in events were noted for acute ischemic stroke (aOR 0.878, 95% CI 0.667–1.156, *p* = 0.354), pericardial effusion (aOR 0.947, 95% CI 0.883–1.016, *p* = 0.129) or cardiogenic shock (aOR 0.761, 95% CI 0.534–1.084, *p* = 0.13). They also had lower odds of in‐hospital mortality (aOR 0.524, 95% CI 0.371–0.74, *p* < 0.001). This data can be seen in Table 2.

**Table 2:** Complications seen in patients aged ≥ 60 with prior long‐term anticoagulants use versus without prior long‐term anticoagulants who underwent LAAO procedures in the United States (post‐PSM analysis).

Complication	*p*‐value	aOR	Lower 95% CI	Upper 95% CI
Cardiogenic shock	0.13	0.761	0.534	1.084
AMI	< 0.001	0.404	0.285	0.573
Death	< 0.001	0.524	0.371	0.74
Sepsis	< 0.001	0.37	0.243	0.564
Intra/postoperative hemorrhage hematoma	0.002	0.757	0.632	0.906
Pericardial effusion	0.129	0.947	0.883	1.016
Cardiac arrest	0.031	0.673	0.47	0.964
Acute kidney injury (AKI)	< 0.001	0.784	0.722	0.851
Acute ischemic stroke	0.354	0.878	0.667	1.156

*Note:* This table presents complications of patients with prior long‐term anticoagulant use versus without prior long‐term anticoagulant who underwent LAAO procedures in the United States (post‐PSM analysis).

Abbreviations: aOR, adjusted odds ratio; CI, confidence interval; LAAO, left atrial appendage occlusion; PSM, propensity score matching.

Pericardial effusion, consistent with other studies, was the most frequent complication, occurring in 2.95% of patients. This was lower than the initial PROTECT AF trial (4.5%) but higher than the subsequent PREVAIL (1.9%) and EWOLUTION trials (0.5%) [3, 4]. Rates we observed would likely be lower today given the updated Watchman FLX 2nd generation and Amulet devices. Device sizing for LAAO is challenging due to variability in LAA size/shape, with oversizing posing a risk for pericardial effusion. Three‐dimensional simulation has the potential to improve this process. Compared to other regularly reported procedural/early post‐procedural complications in the 3 above studies, we find lower rates of hemorrhage (0.45% vs*.* 3.5%, 1.25% and 0.7% respectively) and ischemic stroke (0.2% vs*.* 1.1% and 0.4% respectively) despite a higher mean aged population [4, 5]. In patients without preprocedural long‐term anticoagulation, our study showed a lower mortality rate compared to the LAAO Commission of Evaluation UK Registry (0.2% vs*.* 1%) [5].

Comparing the two groups, patients with long‐term anticoagulation had fewer procedural/in‐hospital complications across most measured domains except for pericardial effusion, cardiogenic shock and ischemic stroke, which were similar between both groups (Tables 1 and 2). Complications in this group may be even lower than reported due to non‐adherence among some patients with AF on long‐term anticoagulation. Long‐term anticoagulation has been shown to reduce systemic inflammation and enhance endothelial function which may explain some of the differences in complications [6]. Additionally, higher use of *peri/post*‐procedural anticoagulation regimens in this group may have contributed to fewer vascular complications compared to the other group. However, some of the differences in complications observed may also be due to the fact that those elderly patients on prior anticoagulation were less sick so better able to tolerate anticoagulation.

This study's strengths include its large sample size from the NIS database undergoing serial data accuracy checks and inclusion of atrial flutter patients which many studies excluded. However, inaccuracies in ICD10 coding are a limitation, while its retrospective nature and lack of ethnic diversity limit its applicability. Specific anticoagulation details and procedural characteristics such as LAAO access site, device type, echocardiographic findings and operator experience were unavailable, posing as potential confounders.

This study demonstrates that preprocedural long‐term anticoagulation is associated with reduced early complications post‐LAAO, except for pericardial effusion, cardiogenic shock and ischemic stroke. These findings suggest that anticoagulation status could influence decisions regarding patient selection, preprocedural anticoagulation management and prognosis determination. Further research should prospectively validate these findings, identify preoperative patient factors for safer outcomes and reassess CHA2DS2‐VA score thresholds for LAAO patients.

## Author Contributions

**Jasninder Singh Dhaliwal:** conceptualization, investigation, writing – original draft, methodology, validation, visualization, writing – review and editing, data curation, formal analysis, project administration. **Samay Mehta:** writing – original draft, writing – review and editing, methodology, conceptualization, visualization, data curation, formal analysis, investigation, validation, project administration. **Manraj S. Sekhon:** methodology, conceptualization, investigation, data curation. **Arush Rajotia:** investigation, validation, writing – review and editing, methodology. **Haresh Gandhi:** formal analysis, data curation, investigation, writing – review and editing. **Kavin Raj:** data curation, methodology, investigation;, validation. **Jarmanjeet Singh:** project administration, writing – review and editing, methodology, investigation. **Mushood Ahmed:** visualization, validation, methodology. **Renuka Verma:** investigation, visualization, validation, methodology. **Hemamalini Sakthivel:** investigation; validation; visualization; methodology. **Kamleshun Ramphul:** formal analysis, data curation, project administration, software. **Raheel Ahmed:** supervision, writing – review and editing, conceptualization, visualization, investigation, methodology, project administration, validation. **Swaiman Singh:** supervision, conceptualization, investigation, writing – review and editing, methodology, visualization, project administration, validation.

## Ethical Statement

The authors have nothing to report.

## Conflicts of Interest

The authors declare no conflicts of interest.

## Transparency Statement

The lead authors, Jasninder Singh Dhaliwal and Samay Mehta, and corresponding author, Raheel Ahmed, affirms that this manuscript is an honest, accurate, and transparent account of the study being reported; that no important aspects of the study have been omitted; and that any discrepancies from the study as planned (and, if relevant, registered) have been explained.

## Data Availability

Patient data available from the National Inpatient Database (NIS), a database maintained by the US government. All data are already fully de‐identified and the data remains available for as long as the NIS database data is maintained. Data that support the findings in this study on the NIS database is accessible after Healthcare Cost and Utilization Project (HCUP) approval.
